# The aluminum distribution and translocation in two citrus species differing in aluminum tolerance

**DOI:** 10.1186/s12870-022-03472-5

**Published:** 2022-03-02

**Authors:** Han Zhang, Xin-yu Li, Mei-lan Lin, Ping-ping Hu, Ning-wei Lai, Zeng-rong Huang, Li-song Chen

**Affiliations:** 1 College of Resources and Environment, Fujian Agriculture and Forestry University, 350002 Fuzhou, China; 2 College of Forestry, Guangxi University, 530004 Nanning, China

**Keywords:** Citrus sinensis, Citrus grandis, Al toxicity, Al distribution, Al translocation, Cell wall

## Abstract

**Background:**

Many citrus orchards of south China suffer from soil acidification, which induces aluminum (Al) toxicity. The Al-immobilization *in vivo* is crucial for Al detoxification. However, the distribution and translocation of excess Al in citrus species are not well understood.

**Results:**

The seedlings of ‘Xuegan’ [*Citrus sinensis* (L.) Osbeck] and ‘Shatianyou’ [*Citrus grandis* (L.) Osbeck], that differ in Al tolerance, were hydroponically treated with a nutrient solution (Control) or supplemented by 1.0 mM Al^3+^ (Al toxicity) for 21 days after three months of pre-culture. The Al distribution at the tissue level of citrus species followed the order: lateral roots > primary roots > leaves > stems. The concentration of Al extracted from the cell wall (CW) of lateral roots was found to be about 8 to 10 times higher than in the lateral roots under Al toxicity, suggesting that the CW was the primary Al-binding site at the subcellular level. Furthermore, the Al distribution in CW components of the lateral roots showed that pectin had the highest affinity for binding Al. The relative expression level of genes directly relevant to Al transport indicated a dominant role of *Cs6g03670.1* and *Cg1g021320.1* in the Al distribution of two citrus species. Compared to *C. grandis*, *C. sinensis* had a significantly higher Al concentration on the CW of lateral roots, whereas remarkably lower Al levels in the leaves and stems. Furthermore, Al translocation revealed by the absorption kinetics of the CW demonstrated that *C. sinensis* had a higher Al retention and stronger Al affinity on the root CW than *C. grandis*. According to the FTIR (Fourier transform infrared spectroscopy) analysis, the Al distribution and translocation might be affected by a modification in the structure and components of the citrus lateral root CW.

**Conclusions:**

A higher Al-retention, mainly attributable to pectin of the root CW, and a lower Al translocation efficiency from roots to shoots contributed to a higher Al tolerance of *C. sinensis* than *C. grandis.* The aluminum distribution and translocation of two citrus species differing in aluminum tolerance were associated with the transcriptional regulation of genes related to Al transport and the structural modification of root CW.

**Supplementary Information:**

The online version contains supplementary material available at 10.1186/s12870-022-03472-5.

## Background

Acidification of arable soils has increased in China from 1980 to 2000 s [1]. Soil acidification significantly accelerates aluminum (Al) solubilization from minerals when the soil pH is less than 5.0 [2]. The excess Al in the soil disturbs the nutrient and water balance of the rhizosphere, thereby reducing crop yield [3], and represents one of the most limiting factors to crop production in tropical and subtropical regions [4]. For instance, it was reported that rice grain yield decreased by 28–62% [5], and wheat grain yield decreased by 23–100% [6] under Al toxicity.

The citrus orchards in south China frequently suffer from Al toxicity induced by soil acidification [7]. For instance, our investigation of 319 soil samples from citrus orchards in Fujian province of China revealed an average pH of 4.34, over 90% of which had a pH less than 5.0 [8]. Under field conditions, excess Al significantly inhibited the root development of *Citrus aurantium* L. [9]. Accordingly, the citrus fruit yield also decreased significantly under Al toxicity [10]. In sandy culture, the biomass of citrus seedlings was depressed by Al toxicity, inducing the oxidative stress and the photosynthetic inhibition of citrus seedlings [11]. Likewise, in the hydroponic culture, high Al concentration induces chlorotic and mottled leaves, thick root tips and less fibrous roots of citrus rootstocks [12].

Plant tolerance to excess Al mainly relies on the inhibition of Al uptake and the restriction of Al translocation [13]. Excess Al accumulates primarily in the roots of citrus seedlings [14, 15]. Furthermore, Al partitioning at the cellular level shows that the plant root CW is the primary location for Al-binding for most crops [16]. The CW was constituted mainly by polysaccharides, such as pectin, cellulose and hemicellulose (HC). However, it is still debatable which CW component contributes most to the Al-binding under high Al concentrations. For instance, Yang et al. [17] reported that HC is the main pool for Al accumulation in *Arabidopsis*. Differentially, Ye et al. [18] proposed that the CW pectin contributed mainly to Al binding in *Panax notoginseng*, a native plant adapted to acid soil. To our knowledge, the Al distribution pattern and the primary Al repository sites in citrus species are still less clear. Moreover, the potential mechanisms regarding the Al distribution and translocation in citrus species are not fully understood and documented.

Genes associated with Al transport and redistribution have been explored. For instance, the gene encoding Al Sensitive 3 (ALS3) has been identified in *Arabidopsis* and is responsible for Al transport from roots to shoots [19]. Moreover, the upregulation of Al-specific transporter Nramp (natural resistance-associated macrophage protein) has been proven to enhance rice Al sensitivity [20]. Our transcriptional study on Al-treated citrus roots also indicated the roles of *Cs3g18690.1* and *Cs6g05460.1* in Al transport and detoxification [21].

We have evaluated the Al tolerance of 12 citrus species and cultivars in 2009 at an AlCl_3_·6H_2_O concentration of 0, 0.2, 0.6, 1.0 and 1.6 mM [22]. The results indicated *C. sinensis* is Al-tolerant and *C. grandis* is an Al-sensitive species. Further studies were carried out to investigate the different responses of *C. sinensis* and *C. grandis* to Al stress at bio-physiological [11, 23], transcriptional [19, 24] and proteomic levels [25, 26]. However, potential Al-tolerant mechanisms in relation to Al distribution and translocation of citrus species are less well understood. In the present study, seedlings of *C. sinensis* (Al-tolerant) and *C. grandis* (Al-sensitive) were cultured by hydroponics using the nutrient solution without Al^3+^ (as a Control) or with 1.0 mM Al^3+^ (as Al toxicity). The Al distribution and translocation were investigated by CW fragmentation to explore the primary Al-binding sites of citrus species. The study also examined the relative expression of genes associated with Al distribution, the kinetics analysis of Al adsorption and desorption, and FTIR analysis of the root CW of two citrus species. The objective of the study is to increase our understanding of the physiological mechanisms underlying the adaptation of citrus species to excessive levels of Al.

## Results

### The Al distribution at the tissue level of citrus species

**Fig. 1:**
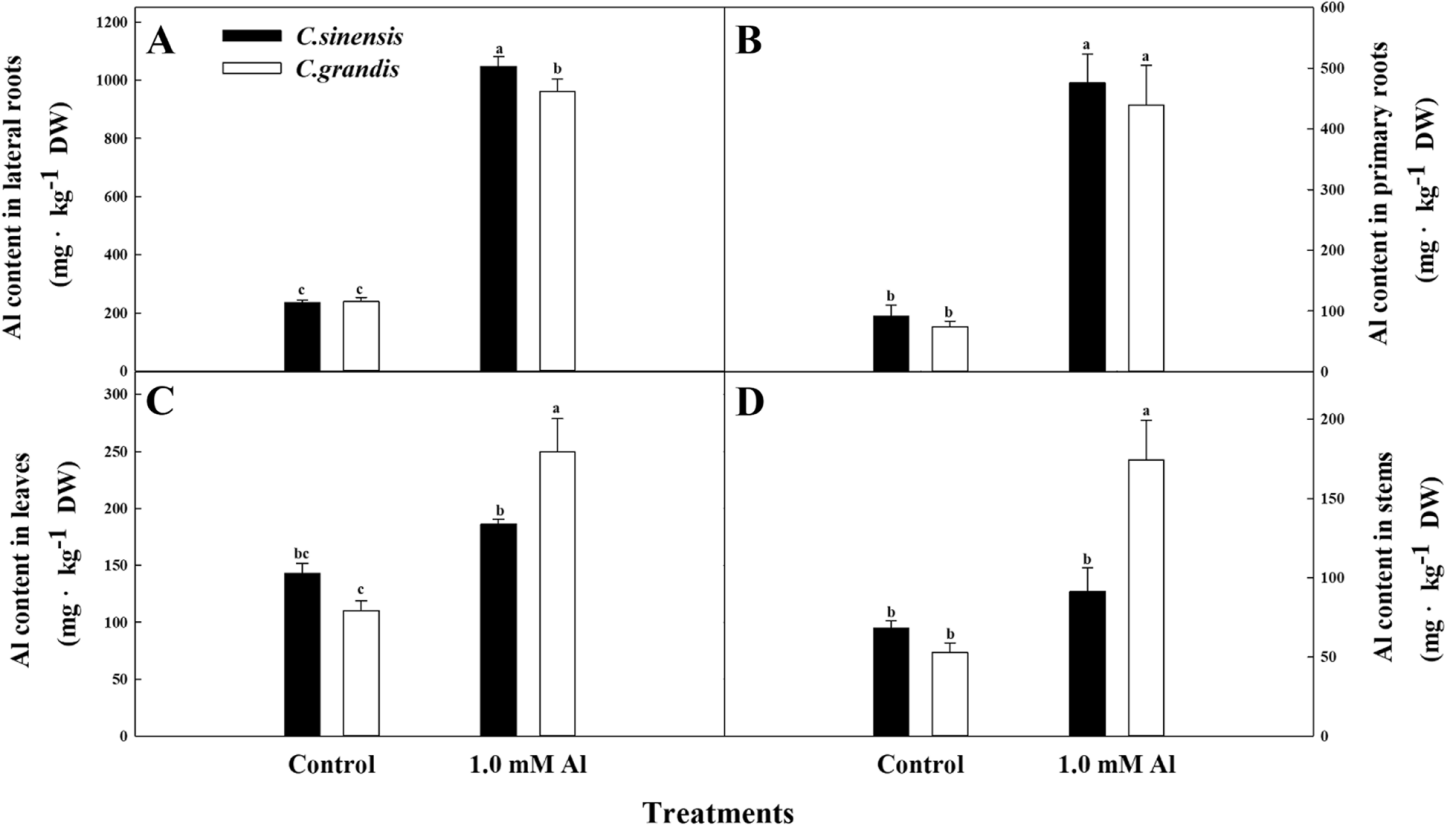
The effects of Al toxicity on Al distribution in lateral roots **A**, primary roots **B**, leaves **C** and stems **D** of *C. sinensis* and *C. grandis* seedlings. Seedlings of *C. sinensis* and *C. grandis* were treated with nutrient solution (Control, pH 4.3) or supplemented by 1.0 mM Al^3+^ (1.0 mM Al toxicity, pH 4.3) for 21 days. The values represent mean ± SE (*N* = 5). Significant differences (*p* ≤ 0.05) between treatments are indicated by different letters

As shown in Fig. 1.0 mM Al treatment significantly increased Al content in lateral roots (Fig. 1a) and primary roots (Fig. 1b) compared to the Control in seedlings of two citrus species. A significant increase of Al content was also found in the Al-treated leaves and the stems in *C. grandis* seedlings (Fig. 1c and d). However, no significant difference in Al content was observed in the Al-treated leaves and stems of *C. sinensis* seedlings compared to the Control. The comparison between citrus species shows that the leaves and stems of *C. grandis* had remarkably higher Al content than *C. sinensis* under Al treatment. In contrast, *C. sinensis* lateral roots had a notably higher Al content than those of *C. grandis* under high Al concentration. Additionally, the Al content at tissue level was found to be lateral roots > primary roots > leaves > stems under Al stress.

### The Al distribution at CW fragments of citrus species

**Fig. 2:**
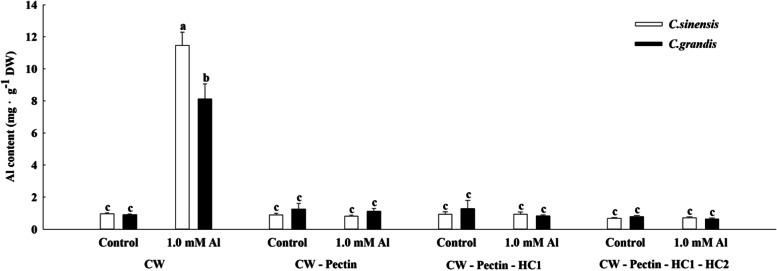
The Al content in different root CW components of *C. sinensis* and *C. grandis* seedlings. Seedlings of *C. sinensis* and *C. grandis* were treated with nutrient solution (Control, pH 4.3) or supplemented by 1.0 mM Al^3+^ (1.0 mM Al toxicity, pH 4.3) for 21 days. Dry lateral roots were used for CW extraction and Al quantification. The values represent mean ± SE (*N* = 5). Significant differences (*p* ≤ 0.05) between treatments are indicated by different letters

The results of Fig. 2 show the Al content significantly increased in the Al-treated root CW of two citrus species compared to Control. The Al level in the root CW was about 8 to 10 times higher than the lateral roots under Al toxicity. Compared to *C. grandis*, *C. sinensis* had a significantly higher Al content in the Al-treated root CW. Differentially, no significant difference of Al content was found among the CW residues after the removal of pectin from the root CW in two citrus species in both the control and the Al toxicity treatment.

### The relative expression of genes associated with Al transport and distribution

**Fig. 3:**
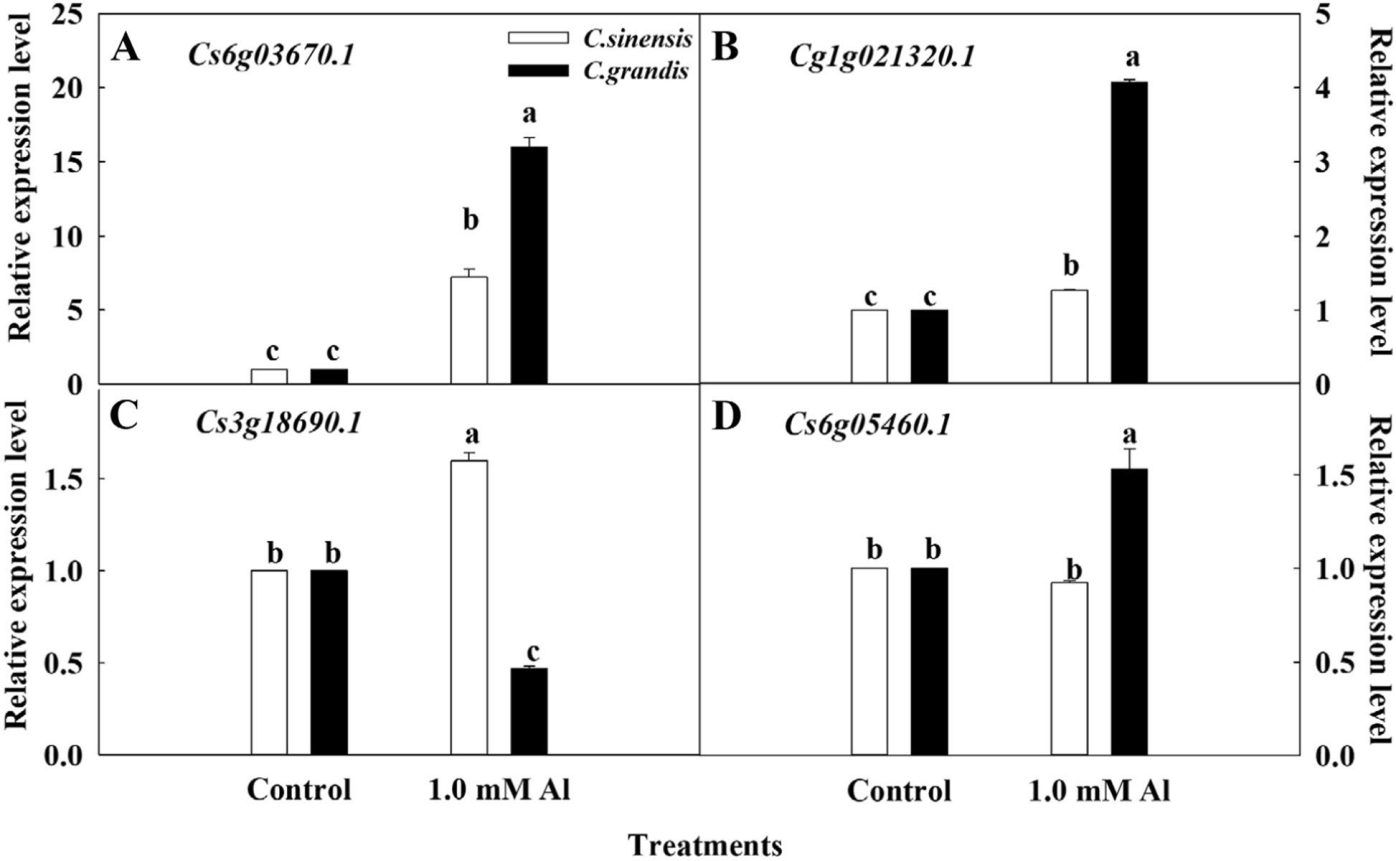
Relative expression levels of *Cs6g03670.1 ***A**, *Cg1g021320.1***B**, *Cs3g18690.1***C** and *Cs6g05460.1***D** in the lateral roots of *C. sinensis* and *C. grandis* seedlings under Al toxicity. Seedlings of *C. sinensis* and *C. grandis* were treated with nutrient solution (Control, pH 4.3) or supplemented by 1.0 mM Al^3+^ (1.0 mM Al toxicity, pH 4.3) for 21 days. The lateral roots of citrus seedlings were harvested for RNA extraction and real-time PCR. The gene relative expression level was normalized to the Control sample. The values represent mean ± SE (*N* = 5). Significant differences (*p* ≤ 0.05) between treatments are indicated by different letters

The relative expression of genes involved in Al transport and distribution of two citrus species was presented in Fig. 3. Compared to the Control, Al toxicity upregulated the expression of *Cs6g03670.1* (3 A) and *Cg1g021320.1* (3B) significantly in roots of two citrus species. Besides, the relative expression level of *Cs6g03670.1* and *Cg1g021320.1* was significantly higher in *C. grandis* compared to *C. sinensis* under Al toxicity. Differentially, the relative expression of *Cs3g18690.1* was upregulated in *C. sinensis* while downregulated remarkably in *C. grandis* (3 C). Compared to Control, no significant difference in the relative expression of *Cs6g05460.1* was found in Al-treated roots of *C. sinensis*. However, the Al toxicity upregulated the relative expression *Cs6g05460.1* in *C. grandis* roots compared to Control (3D).

### The Al adsorption and desorption analysis of root CW of citrus species

**Fig. 4:**
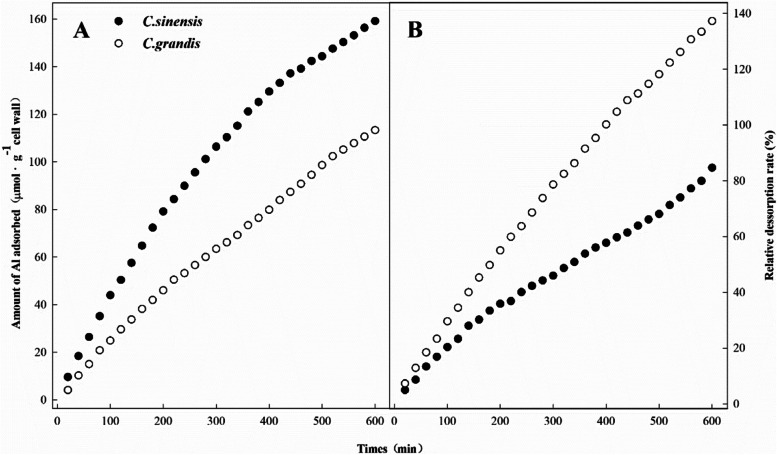
The Al Adsorption **A** and desorption **B** kinetics of lateral root CW of *C. sinensis* and *C. grandis*. Seedlings of *C. sinensis* and *C. grandis* were treated with nutrient solution (Control, pH 4.3) for 21 days. The lateral root CW samples were extracted for Al adsorption and desorption kinetics analysis within 600 min

The CW of the lateral root of two citrus species was extracted for kinetics analysis of Al adsorption and desorption within 600 min. As shown in Fig. 4a, Al uptake in the root CW of *C. sinensis* was higher in comparison to *C. grandis*, indicating the root CW of *C. sinensis* had a higher capacity for Al binding than *C. grandis*. Moreover, the relative desorption rate in Fig. 4b showed that the lateral root CW of *C. sinensis* had a higher Al-binding rate than that of *C. grandis*. By comparison, *C. sinensis* had a lower Al desorption rate compared to *C. grandis* up to 600 min.

### The FTIR spectra of lateral root CW

**Fig. 5:**
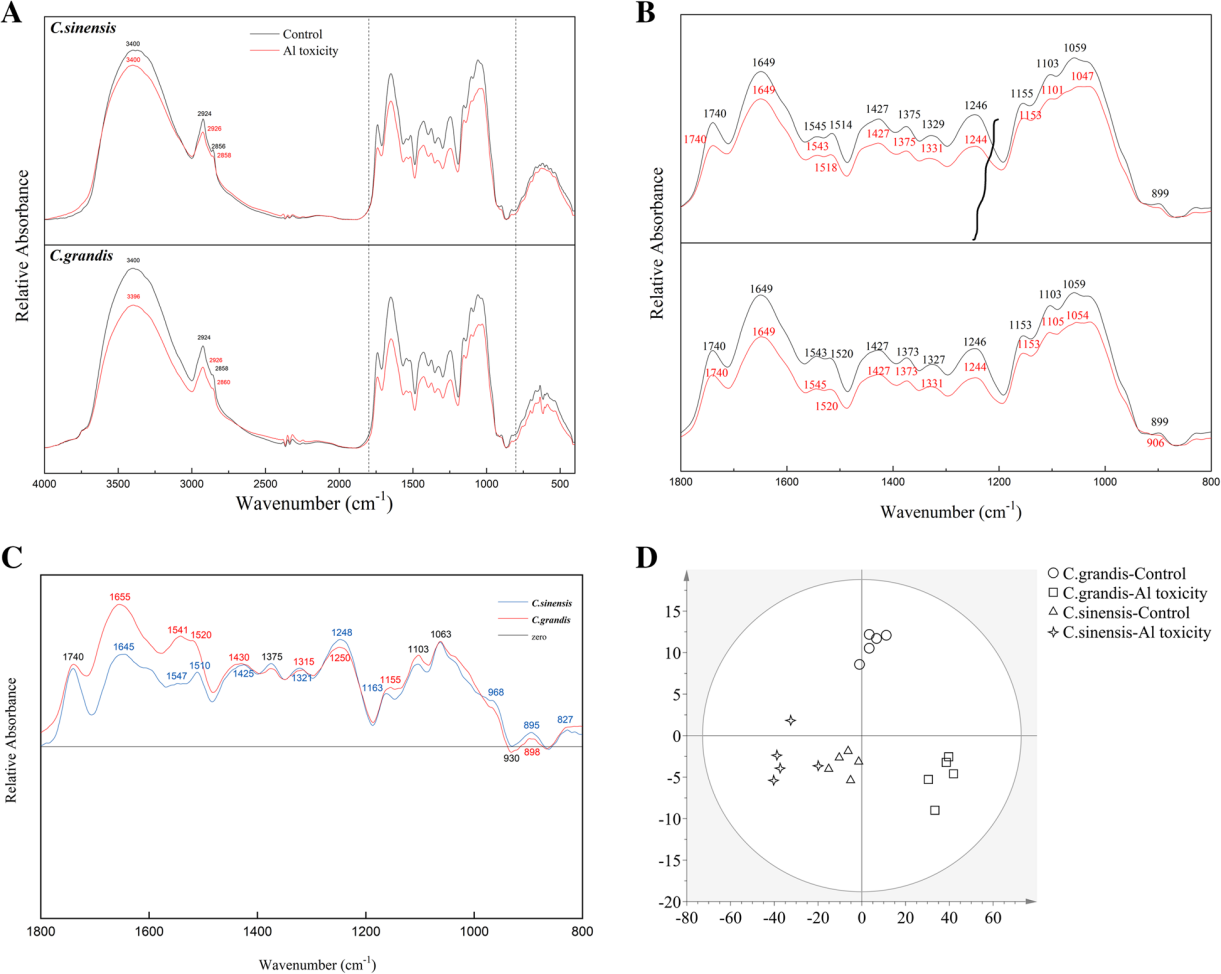
The FTIR spectra of the lateral root CW in the region of 4000–500 cm^− 1^ **A**, 1800–800 cm^− 1^ **B**, digital subtraction spectra **C** and the OPLS-DA of relative absorbance **D** of two *c*itrus species. Seedlings of *C. sinensis* and *C. grandis* were treated with nutrient solution (Control, pH 4.3) or supplemented by 1.0 mM Al^3+^ (1.0 mM Al toxicity, pH 4.3). The lateral root CW samples were extracted for FTIR spectra analysis. The digital spectra represent Control CW minus Al toxic-CW. The values represent mean ± SE (*N* = 5)

The alterations of CW composition and structure by Al toxicity were revealed by FTIR analysis (Fig. 5). The wavenumber of spectra from two citrus species and related assignments were listed in Table 1. The results show that the root CW of the Control from two citrus species had almost the same band position, indicating the similar composition of chemical groups of the root CW. However, band positions shifted under Al toxicity differentially in two citrus species. For instance, the vibration located at 3400 cm^− 1^ was moved to 3396 cm^− 1^ in *C. grandis* by Al toxicity. The vibration was shifted from 2856 cm^− 1^ to 2858 cm^− 1^ in *C. sinensis* and 2858 cm^− 1^ to 2860 cm^− 1^ in *C. grandis* by Al toxicity. It was also strikingly to find that most band positions from 1800 cm^− 1^ to 800 cm^− 1^, associated with polysaccharides, amide and ester, were shifted in both of two citrus species by Al toxicity. Apart from the band position shift, the relative absorbance at most band positions was also decreased by Al toxicity in two citrus species compared to the Control (Fig. 5a and b). The digital subtraction spectra were generated by subtracting the Al-treated spectra from the Control spectra of the CW of two citrus species, respectively. As found in Fig. 5c, the band intensity of the *C. grandis* was stronger than that of *C. grandis* overall. The OPLS-DA (orthogonal partial least-squares discrimination analysis) on the relative absorbance also reflected a more apparent separation between the Control and Al-toxic CW of *C. grandis* than *C. sinensis* (Fig. 5d).

**Table 1:** The infrared absorption frequencies of the CW and tentative assignment. Seedlings of *C. sinensis* and *C. grandis* were treated with nutrient solution (Control, pH 4.3) or supplemented by 1.0 mM Al^3+^ (1.0 mM Al toxicity, pH 4.3). The lateral root CW samples were extracted for FTIR spectra analysis

Wavenumber (cm^-1^)				**Tentative Assignment**	**Reference**
***C. sinensis***		***C. grandis***			
**Control**	**Al toxicity**	**Control**	**Al toxicity**		
3400	3400	3400	3396	OH stretching	[27]
2924	2926	2924	2926	CH asymmetric stretching	[28]
2856	2858	2858	2860	CH symmetric stretching	[28]
1740	1740	1740	1740	C=O stretching of ester	[28]
1649	1649	1649	1649	C=O stretching of amide I band	[29]
1545	1543	1543	1543	NH bending and CN stretching of amide II band	[29]
1514	1518	1520	1520	NH bending and CN stretching of amide II band	[30]
1427	1427	1427	1427	CH2 symmetric deformation	[31]
1375	1375	1373	1373	COO- symmetric stretching and aliphatic group vibration	[32]
1329	1331	1327	1331	C-O	[33]
1246	1244	1246	1244	C=O stretching or NH bending of amide III bands	[32]
1155	1153	1153	1153	phosphoryl group	[28]
1103	1101	1103	1105	C=O stretching, alclhol hydroxyl, ether or ester base	[29]
1059	1047	1059	1054	C-OH stretching of alcoholic groups and carboxylic acids	[34]
899	-	899	906	β-linkage between two glucose units	[31]

## Discussion

Citrus fruit trees are superbly adapted to the acid soils with potentially high concentrations in south China [21]. Understanding of Al partition and mobilization *in vivo* is pivotal to revealing the mechanism of Al tolerance of citrus species. Moreover, discerning Al binding sites in citrus species is of great significance in the development of Al mitigation strategies. The present study addressed these challenges. Hydroponic culture has been widely used to explore the ion behavior of citrus species [35, 36]. Compared to our previous study in sandy culture with a 1.0 mM Al treatment for 18 weeks [23], the present 21 days’ hydroponic culture of citrus species resulted in almost the same Al level in leaves, indicating a reliable treatment for this study.

It has been shown that Al-induced phytotoxicity has many target sites from the apoplast to symplast in higher plants [37]. Accordingly, plant species vary in Al-tolerance and have evolved different strategies to cope with Al toxicity based on Al distribution and translocation. Plant species native to acid soils are often found to retain excess Al in insensitive roots, protecting leaves from metabolic disruption [38]. For instance, Kopittke et al. [13] reported that the Al-tolerant wheat accumulated more than four times of Al in roots compared to the sensitive line. Similarly, the higher Al content on the root apex was also observed in Al-tolerant common bean compared to an Al-sensitive genotype [39]. The present results support a greater accumulation of Al in roots under Al stress compared to shoots in citrus species (Fig. 1). Furthermore, the results also demonstrated that a significantly higher Al storage in lateral roots but significantly lower Al content transported to the shoots in *C. sinensis* compared to *C. grandis* under Al stress. Likewise, the higher Al translocation of *C. grandis* than *C. sinensis* was also found in 1.0 mM Al-treated citrus seedlings under 18 weeks’ sandy culture [11]. The relative expression level of genes directly involved in Al transport of citrus species indicated lower expression of *Cs6g03670.1*(*ALS3*) and *Cg1g021320.1*(*Nramp6*) could result in less Al transport from roots to shoots in *C. sinensis* compared to *C. grandis* (Fig. 3 A and 3B), which was in line with our previous investigation [40]. However, the Al stress within 21 days in the present study did not significantly affect the biomass accumulation of two citrus species (data not shown). With the stress duration increased to 15 weeks, the *C. sinensis* seedlings had remarkably higher biomass accumulation than *C. grandis* in both leaves and roots (Additional file 1: Figure S1). Conclusively, the relatively higher Al tolerance of *C. sinensis* is related to less Al translocation from the roots to shoots.

The plant root CW is the first defense against Al toxicity. Clarkson et al. [41] revealed that over 85% of Al accumulated on the CW of barley roots, and for woody plants, more than 88% of total Al was localized in the root CW of the conifer [42]. In the present study, the CW of citrus lateral roots accumulates about 8 to 10 times higher Al content than lateral roots, indicating the prominent roles of the root CW in Al immobilization of citrus species. Interestingly, the ratio of CW-binded Al is very close to the finding of Al-treated tea (*Camellia sinensis*) roots [43]. Moreover, the comparison of Al distribution in CW fractions suggests pectin has the greatest affinity for Al of CW polysaccharides in citrus roots. The contribution of pectin in Al sequestration was also reported in rice roots [44]. Li et al. [45] proposed that a high density of carboxylic groups on the pectin contributes to Al binding. Further studies regarding pectin content and related structural deformation of roots under Al stress of citrus species are needed to reveal the role of pectin in Al detoxification.

Ma et al. [46] reported that the CW of Al sensitive wheat had higher Al retention than Al tolerant cultivar under 10 µM Al within a 9 h exposure time. By contrast, we observed that a remarkably higher Al content in the CW of *C. sinensis* lateral roots than that of *C. grandis* (Fig. 2), which is consistent with the higher Al content of lateral roots in *C. sinensis* than *C. grandis*. Therefore, we propose that the Al distribution pattern in higher plants depends on the toxic intensity, such as Al level and stress duration. For example, the Al tolerant cultivar exclude Al encountering weak Al stress, which resulted in less Al accumulation. However, when the Al exclusion is not enough for Al detoxification, the excessive Al will be transported and redistributed *in vivo*, such as Al stabilization on the roots or CW.

The adsorption and desorption kinetics demonstrated that the root CW of *C. sinensis*, an Al-tolerant species, had a higher Al affinity than *C. grandis* (Fig. 4). By contrast, the root CW of *C. grandis* exhibited a lower Al adsorption and a higher Al desorption, indicating less tight Al-binding on the root CW, which would facilitate higher Al translocation from apoplast to symplast. Therefore, we infer that Al-tolerant woody plants tend to retain excess Al on the root CW to diminish Al translocation owing to their high retention capacity of the root systems. Besides, the Al binding firmly on roots is economical for Al resistance considering the energy cost during Al translocation. The findings of the present study also implied that organic material prepared from CWs is promising in alleviating the Al toxicity of the citrus plants in acidic red soils.

The Al binding resulted in modification of the root CW, which could be assessed by FTIR analysis [47, 48]. The results of this study show that almost no new characteristic peaks emerged indicating less effect of Al toxicity on the types of functional groups on the CW by Al toxicity overall in two citrus species. The modification of CW by Al stress is mainly dependent on the abundance of chemical groups on the root CW of citrus species. For instance, the spectra at 3400 cm^− 1^ (-OH stretching), was shifted to 3396 cm^− 1^ under Al stress in *C. grandis*, suggesting the changes in hydrogen-bonding mode and the damaging of connections between CW components by Al toxicity (Table 1). The results support less Al tolerance of *C. grandis* than *C. sinensis* by considering the flexible deformation of hydrogen bonds between molecules [26]. Also, a previous study has shown that the absorbance at 1740 and 1649 cm^− 1^ represents the absorption of the esterified and non-esterified carboxyl groups of pectin, respectively [49]. The present results of downregulated relative absorbance at 1740 cm^− 1^ and 1649 cm^− 1^ were coincident with significantly higher Al accumulation in the pectin (Fig. 5c), suggesting the role of CW pectin in Al-binding under Al toxicity. Besides, it is also interesting to find that the vibrations from 1200 cm^− 1^ to 900 cm^− 1^ (Table 1), which belong to the polysaccharide fingerprint region [50], shifted and decreased under Al toxicity. The results indicated that the altered structure and content of CW polysaccharides under Al toxicity would affect the Al binding on the root of citrus species. Both of the digital subtraction spectra (Fig. 5c) and the OPLS-DA (Fig. 5c) of relative absorbance in two citrus species supported a greater alteration of the CW in *C. grandis* compared to *C. sinensis*, such as more severe damage under Al toxicity. Similarly, a higher relative absorbance of the upper leaves corresponding to a much obvious symptom of boron deficient orange seedlings compared to lower leaves has also been reported based on the FTIR analysis [35]. Further studies based on isotope labeling of Al and pectin deformation and polysaccharides quantification of the citrus root CW are needed to disclose the Al spatial and temporal distribution of Al.

## Conclusions

At the tissue level, citrus lateral roots were the primary Al-binding site under Al toxicity. At the subcellular level, the pectin of the CW was most abundant in the Al-accumulation of citrus species. Compared to *C. grandis*, a less tolerant citrus species, *C. sinensis* had a higher Al retention on the root CW and a lower Al translocation efficiency from roots to shoots upon exposure to toxic levels of Al (1.0 mM Al). Both the transcriptional regulation of genes related to Al transport and the structural modification of root CW contributed to the Al translocation of two citrus species. Future investigations on the Al partition and translocation mediated by CW modification are necessary to fully elucidate the mechanisms of Al tolerance in citrus species.

## Methods

### Plant culture and treatments

The citrus species ‘Xuegan’ [*Citrus sinensis* (L.) Osbeck] and ‘Suanyou’ [Citrus grandis (L.) Osbeck] used in the study were identified by Professor Lin-tong Yang of Fujian Agriculture and Forestry University (Fuzhou, China) and deposited as living materials for research purposes in the demonstration orchard of Fujian Academy of Forestry Sciences (FAFS). In December 2018, citrus fruits were harvested under the permission of Professor Xiang-xi Xiao in FAFS and stored in a fridge at 4 ℃. For germination, the seeds of *C. sinensis* and *C. grandis* were sown in a plastic tray filled with clean river sand at the greenhouse in early April of 2019. Four weeks after germination, seedlings of uniform size (about 10 cm) were transferred to black tanks containing nutrient solution and aerated for 30 min every two hours. The nutrient solution contained 1 mM KNO_3_, 1 mM Ca(NO_3_)_2_, 0.1 mM KH_2_PO_4_, 0.5 mM MgSO_4_, 10 µM H_3_BO_3_, 2 µM MnCl_2_, 2 µM ZnSO_4_, 0.5 µM CuSO_4_, 0.065 µM (NH_4_)Mo_7_O_24_ and 20 µM Fe-EDTA. The pH of the nutrient solution was adjusted to 4.30 using 1 M HCl or NaOH and was replaced every two days. Three months after transplanting, the plants were subjected to the treatments with 0 (Control) or 1.0 mM Al (Al toxicity) in the nutrient solution described above (pH 4.30). The samples of citrus leaves, stems, primary roots and lateral roots were divided and collected 21 days after treatments when visible leaf chlorosis appeared on Al-treated *C. grandis* leaves.

### Quantification of Al at the tissue level

The leaves, stems, primary roots and lateral roots of citrus species were dried and digested in HNO_3_/HClO_4_ (5:1, v/v), and Al content was quantified according to Hsu [51].

### Quantification of Al on CW fractions of citrus lateral roots

The crude CW of citrus lateral roots was extracted according to Zhong and Lauchli [52] with modifications. Briefly, about 50 mg citrus lateral root was powdered and polled into a centrifuge tube with 5 mL ice-cold 75% ethanol for 20 min on ice. The samples were then centrifuged at 1000 g for 10 min. The supernatant was discarded, and the resulting pellets were centrifugated at 17,000 g for 10 min three times with 5 mL 80% ethanol, methanol-chloroform mixture (1:1, v/v) and acetone, respectively. The final pellets were pooled as crude CW after being dried and weighed.

The dry crude CW from the lateral roots of citrus seedlings was further fractioned according to Yang et al. [17] Briefly, the dry crude CW was added into ammonium oxalate (containing 0.1% NaBH_4_, pH = 4.0) (5 mg CW/1mL solution) in a boiling water bath for one hour and centrifuged at 17,000 g for 10 min for three times to remove the pectin, the resulting pellet (CW-pectin) was pooled and dried. The CW-pectin fraction was extracted by 4% KOH (containing 0.1% NaBH_4_) or 24% KOH (containing 0.1% NaBH_4_) under room temperature three times for 24 h in total to further remove the hemicellulose 1 (HC-1) and hemicellulose 2 (HC-2). The pooled pellets were CW-pectin fractions without HC1 (CW-pectin-HC1) and HC2 (CW-pectin-HC1-HC2), respectively. The Al content of the CW and CW fractions (CW-pectin, CW-pectin-HC1 and CW-pectin-HC1-HC2) was quantified according to the method described above. The Al content of CW and CW fractions was expressed as mg · g^− 1^ DW (dry weight) of lateral roots.

### The relative expression analysis of genes involved in Al translocation

Lateral roots of two citrus species were harvested for the relative expression analysis of genes (*Cs6g03670.1*, *Cg1g021320.1*, *Cs3g18690.1* and *Cs6g05460.1*) involved in Al translocation according to Wu et al. [40]. Briefly, total RNA in lateral roots was extracted using TRIzol reagent (Invitrogen, Carlsbad, CA, USA). The Fastking FIRST STRAND cDNA synthesis kit (Tiangen, Beijing, China) was used for cDNA synthesis following the manufacturer’s instructions. RT-qPCR was performed using 2 RealStar Green Fast Mixture (Genstar, China) in CFX96™ Real-Time System according to the manufacturer’s instructions. The 20 µL reaction system contained 10 µL iQ SYBR green supermix, 0.5 µL each of 10 µM primers, 1.0 µL (5 ng) DNA template, and 8.0 µL RNAase-free H_2_O. The program consisted of initial denaturation at 95 ^◦^C for 2 min, followed by 40 cycles of 95 ^◦^C for 10 s, 57 ^◦^C for 35 s and 72 ^◦^C for 30 s. Citrus genes actin (*Cs1g05000.1*) was used as internal references [53]. The primers were designed using Primer Premier 5 software (Premier Biosoft Ltd., Palo Alto, CA, USA) based on the gene sequences from Citrus Genome Database (https://www.citrusgenomedb.org/) and listed in Table 2. The data were processed by the method of 2^−ΔΔCT^ according to Livak and Schmittgen [54]. The gene relative expression was normalized to Control. The fold changes of each gene were used for statistical analysis. There were 4 biological replications with 3 technical replicates of each.

**Table 2:** Genes and their primers for relative expression analysis using RT-qPCR

Accession number	Description	Forward (F) and reverse (R) primer sequences
*Cs6g03670.1*	Aluminum Sensitive 3 (ALS3)	F: 5’ TGCTGCTGGCTGTCCTGTT 3’ R: 5’ TGCTTTGTTGCCTGTCTCG 3’
*Cg1g021320.1*	Metal transporter Nramp6	F: 5’ TAACTGGAACTTATGCGGGACA 3’ R: 5’ CTGCCATTGCCGAAAAC 3’
*Cs3g18690.1*	Heavy metal transport/detoxification superfamily protein	F: 5’ GTGGACTTGAAGCAGCAGAA 3’ R: 5’ TGAGCACGCATTAGGATTTT 3’
*Cs6g05460.1*	Heavy metal transport/detoxification superfamily protein	F: 5’ TACCCTCTGCCCCTTGTTC 3’ R: 5’ GCTAATGGCTTGGAGTTGGAT 3’
*Cs1g05000.1*	Reference gene as internal control	F: 5’ TTTACCACCACAGCCGAACG 3’ R: 5’ TGGAGCCACGACCTTGAT 3’

### Al adsorption and desorption kinetics

The Al adsorption and desorption kinetics were performed according to Zheng et al. [55] with modifications. Briefly, the adsorption solution of 0.5 mM Al^3+^ in 0.5 mM CaCl_2_ (pH 4.30) was pumped by a peristaltic pump at 0.2 mL/min through a 2 mL column loaded with 10 mg root CW for Al adsorption. The solution after CW adsorption was then collected by a fraction collector at 20 min intervals until the Al content was equal to the adsorption solution. The residue Al^3+^ left in the system was washed by 0.5 mM CaCl_2_ (pH 4.5) at 0.6 mL/h for 1 h before Al desorption by 2.5 mM CaCl_2_ (pH 4.30) at 0.2 mL/h until the Al concentration in the collector was below the detection limit. Finally, the Al content in the fraction collector was quantified, and the kinetics were analyzed within 600 min. The Al absorption and desorption kinetics were performed three times independently.

### FTIR spectra analysis

2 mg dry CW of citrus lateral root was mixed with 200 mg KBr and pressed into a disk by FW-5 A Pressor. The IR spectra of CWs ranging from 4000 − 400 cm^-1^ were recorded using Vertex 70 spectrometer with a resolution of 4 cm^-1^ and 32 scans per sample. The obtained spectra were normalized and baseline-corrected by OPUS management software before being exported to Excel. The data of FTIR spectra were processed by Origin Pro 2020b (OriginLab Corporation, USA). The OPLS-DA was performed in SIMCA 14.1 (Umetrics AB, Umea, Sweden).

### Data analysis

Data analysis was performed by two-way analysis of variance, and significant differences (*P* < 0.05) among treatments were statistically evaluated by two-way ANOVA using Duncan’s test, using the SPSS 16.0 (SPSS Corp., Chicago, IL, USA). All the values are presented as means ± SE. Figures except OPLS-DA were generated by using Sigmaplot 12.0.

## Supplementary Information

**Additional file 1: Figure S1. **The effects of Al toxicity on the DWs of whole plants (A), roots (B), stems (C), leaves (D), shoots (E) and ratio of root/shoot (F) of *C. sinensis* and *C. grandis* seedlings. Seedlings of *C. sinensis* and *C. grandis* were treated with nutrient solution (Control, pH 4.3) or supplemented by 0.5, 1.0 and 2.0 mM Al^3+^ (pH 4.3) for 15 weeks. The values represent mean ± SE (*N* = 6). Significant differences (*p* ≤ 0.05) between treatments are indicated by different letters.

## Data Availability

The DNA sequences are accessible in Citrus Genome Database (https://www.citrusgenomedb.org/). All data analyzed in this study are included in this published article and its additional files.

## Abbreviations

ALS3: Al sensitive 3
CW: cell wall
DW: dry weight
FTIR: Fourier transform infrared spectroscopy
HC: hemicellulose
Nramp: natural resistance-associated macrophage protein
OPLS-DA: orthogonal partial least-squares discrimination analysis
