# Supplementary figures and images for: The aluminum distribution and translocation in two citrus species differing in aluminum tolerance
Source: BMC Plant Biol. 2022 Mar 2;22:93. doi: 10.1186/s12870-022-03472-5 (PMC8889769; doi:10.1186/s12870-022-03472-5)

Figure S1


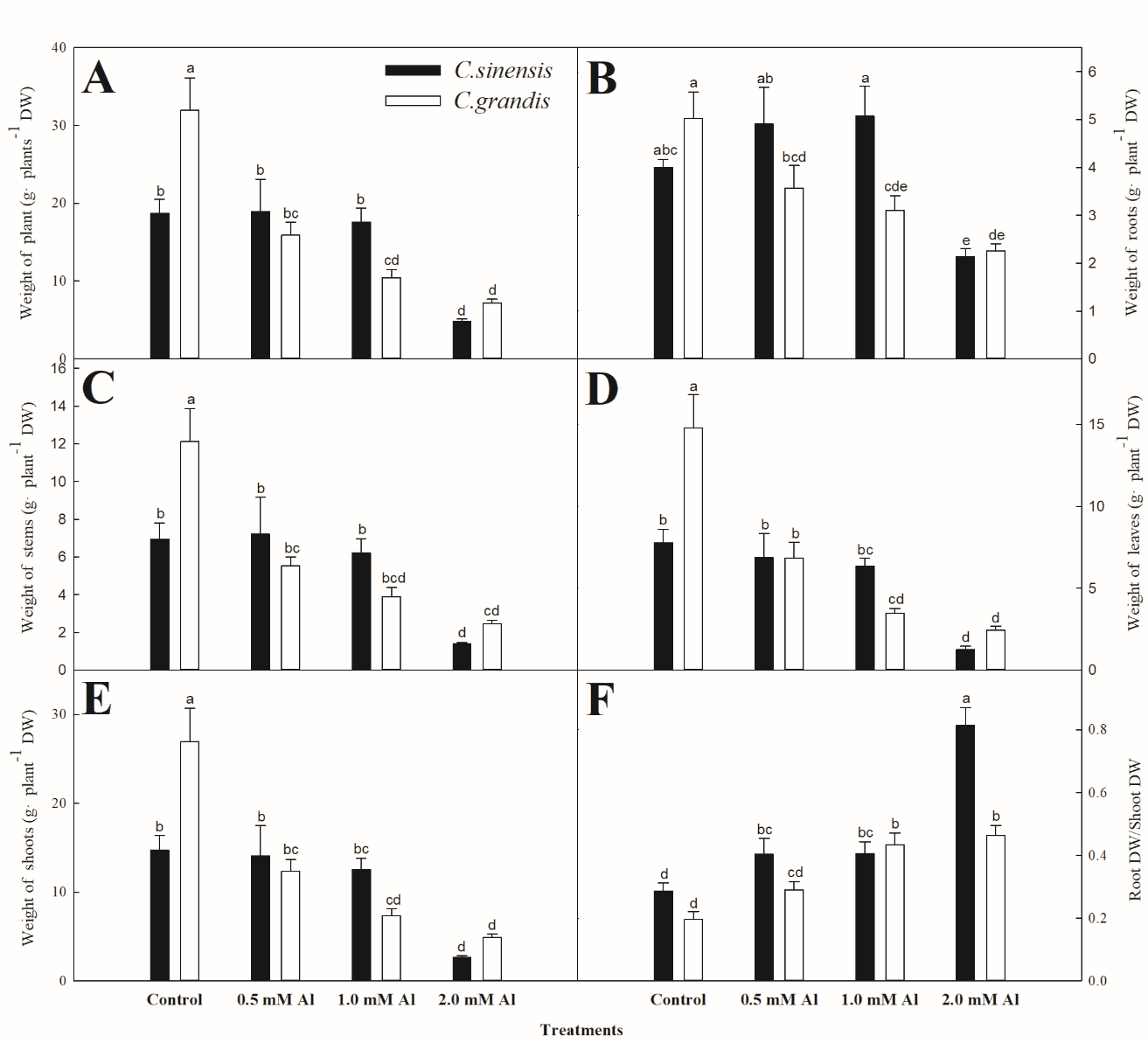

Supplement: Supplementary file 1 — Additional file 1: Figure S1. The effects of Al toxicity on the DWs of whole plants (A), roots (B), stems (C), leaves (D), shoots (E) and ratio of root/shoot (F) of C. sinensis and C. grandis seedlings. Seedlings of C. sinensis and C. grandis were treated with nutrient solution (Control, pH 4.3) or supplemented by 0.5, 1.0 and 2.0 mM Al3+ (pH 4.3) for 15 weeks. The values represent mean ± SE (N = 6). Significant differences (p ≤ 0.05) between treatments are indicated by different letters. [file 12870_2022_3472_MOESM1_ESM.docx]
